# Transgelins: Cytoskeletal Associated Proteins Implicated in the Metastasis of Colorectal Cancer

**DOI:** 10.3389/fcell.2020.573859

**Published:** 2020-10-07

**Authors:** Jingwen Liu, Yingru Zhang, Qi Li, Yan Wang

**Affiliations:** ^1^Department of Medical Oncology, Shuguang Hospital, Shanghai University of Traditional Chinese Medicine, Shanghai, China; ^2^Academy of Integrative Medicine, Shanghai University of Traditional Chinese Medicine, Shanghai, China

**Keywords:** transgelins, colorectal cancer, metastasis, signaling pathways, molecular targets, calponin homology domain

## Abstract

Transgelins, including transgelin-1 (T-1), transgelin-2 (T-2), and transgelin-3 (T-3), are a family of actin-binding proteins (ABPs) that can alter the structure and morphology of the cytoskeleton. These proteins function by regulating migration, proliferation and apoptosis in many different cancers. Several studies have shown that in various types of tumor cells, including colorectal cancer (CRC) cells, and in the tumor microenvironment, the expression and biological effects of transgelins are diverse and may transform during tumor progression. Previous researches have demonstrated that transgelin levels are positively correlated with metastasis in CRC, and down-regulating their expression can inhibit this process. In advanced disease, T-1 is a tumor activator with increasing expression, and T-2 expression increases with the progression of CRC. Finally, T-3 is only expressed in neurons and is not associated with CRC. This evidence suggests that T-1 and T-2 are potential biomarkers and therapeutic targets for CRC metastasis.

## Introduction

Colorectal cancer (CRC) is one of the leading causes of cancer death worldwide, and because of its increasing incidence in younger populations coinciding with a decreasing incidence in older individuals, the CRC patient population as a whole is rapidly shifting toward a younger demographic (Siegel et al., 2020). CRC is generally asymptomatic until it reaches an advanced stage, and more than 25% of CRC patients have lymph node or even distant metastasis at initial diagnosis (stage IV) (Tauriello et al., 2017; Sterpetti et al., 2020). Metastasis is the main cause of death in patients with CRC (Van Cutsem et al., 2018; Hafizi et al., 2019), among which liver and lung metastasis account for approximately 60 and 30% of these deaths, respectively (Wang et al., 2018; Li et al., 2019; Kasprzak and Adamek, 2020). CRC metastasis is a multihit, multistage process, but there is a lack of effective treatments for CRC metastasis (Wei et al., 2019); therefore, the identification of molecular biomarkers associated with CRC metastasis is critical for the subsequent treatment of CRC and extending patient survival.

Transgelins are a family of proteins that were discovered decades ago (Lees-Miller et al., 1987; Shapland et al., 1993). They are involved in many diseases, including asthma, hypertension, diabetes and cancer, but the role of transgelins in cancer is not very clear (Huang et al., 2018; Sun et al., 2018; Varberg et al., 2018; Yin et al., 2018). Recently, studies have shown that transgelins are associated with CRC metastasis. Mo et al. (2020) conducted weighted gene co-expression network analysis (WGCNA) and found that transgelins may contribute to the development of early onset CRC; further experiments identified transgelin 1 (T-1) as the top-ranked biomarker of node status. T-1 was up-regulated in node-positive CRC compared with node-negative disease (Lin et al., 2009). Almeida et al. (2017) discovered that tumor tissues from CRC patients presented several up-regulated proteins that are linked to cytoskeletal stability and cell migration, such as actin-binding proteins (ABPs), transgelin, and so on. However, the role of transgelins in cancer remains unclear and controversial because some reports have shown the loss of transgelin expression during CRC progression and tumor suppressor activity (Shields et al., 2002; Yeo et al., 2010; Chunhua et al., 2013). T-2, a homolog of T-1, is highly expressed in CRC, suggesting that it is a potential biomarker to estimate the progression and prognosis of CRC (Zhang et al., 2010; Cheshomi and Matin, 2018; Yin et al., 2019). Members of the transgelin family have the potential to alter the motility, adhesion, and morphology of cells by directly interacting with the actin cytoskeleton, which leads to cell migration, proliferation, and apoptosis (Lin et al., 2009; Chen Z. et al., 2019). Based on this evidence, we considered that transgelins could be a potential target for treating CRC metastasis.

## Molecular Biological Characteristics

**FIGURE 1:**
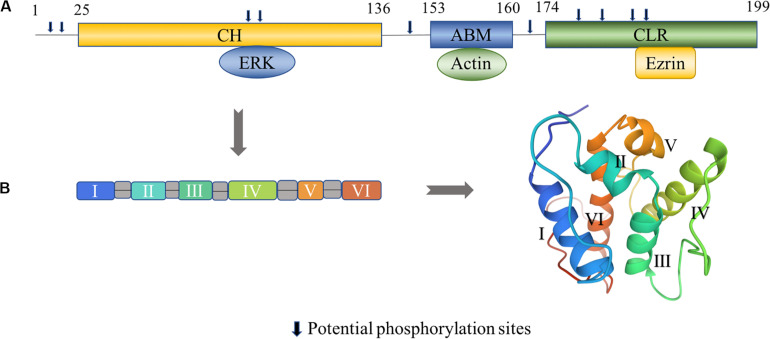
Structural characteristics of transgelin. **(A)** Human transgelin consists of three regions: an N-terminal calponin-homolog (CH)-domain, an actin-binding motif (ABM), and a C-terminal calponin-like repeated (CLR)-region. The CH domain binds to ERK, the ABM domain binds to actin, and the C-terminal CLR domain binds to ezrin (Yin et al., 2019). Potential phosphorylation sites amino acids number 8, 11, 83, 84, 145, 163, 180, 185, 190, and 192 in T-2. **(B)** The tertiary fold of the CH domain (PDB: 1H67). The CH domain contains six α-helices, in which two short helical structures (II and V) and a core of four α-helices (I, III, IV, and VI) are present. Helices III and VI are approximately parallel, while helix IV is lying oblique to the other helices (Yin et al., 2020). The 3D view of the structural model was generated from the data file from the Protein Data Bank.

Transgelin, a 22 kDa protein, was first discovered in chicken gizzard smooth muscle and named for its ability to bind actin (Lees-Miller et al., 1987; Shapland et al., 1993; Li et al., 2008). There are three isoforms in the transgelin family based on the isoelectric point: T-1 (SM22α), T-2 (SM22β), and T-3 (SM22γ) (Carboni et al., 2006; Dowd et al., 2008; Fukushima et al., 2011), and they differ in cell type expression (Table 1). As a member of the calponin protein family, transgelin contains a single C-terminal calponin-like module (CLIK23) and an N-terminal calponin-homolog (CH) domain comprising six α-helices that interact with the actin complex (Lawson et al., 1997; Fu et al., 2000; Li et al., 2008; Figure 1B). FRAP analysis shows that CLIK23 supports the stabilized interaction between actin and the type-3 CH-domain-mediated interaction of it (Matsui et al., 2018). Transgelin, with its type-3 CH domain, binds to actin upon interacting with extracellular signal-regulated kinase (ERK) (Figure 1A).

**TABLE 1:** Physicochemical property of human transgelins.

Isoforms	Chromosome location	Exons	Transcripts	Length (*aa)	Identity to T1 (%)	Isoelectric	Tissue expression	References
T-1	11q23.3	5	8	201	100	9.0	Visceral and vascular smooth muscle cells	Camoretti-Mercado et al., 1998; Kersey et al., 2018; Zerbino et al., 2018
T-2	1q23.2	7	5	199/220	64.7	8.4	Smooth muscle cells and the immune system	Kim et al., 2017; Jo et al., 2018; Yin et al., 2019

*aa, Amino acid.

### Characteristics of T-1

T-1 is also known as SM22α(Lees-Miller et al., 1987) and WS3-10 (Murano et al., 1991) and is a homolog of mouse p27 (Almendral et al., 1989). Its encoding gene (TAGLN1) is localized to chromosome 11q23.2, with an isoelectric point of 9.0 and a length of 5.4 kb (Camoretti-Mercado et al., 1998), containing five exons and four introns; these five exons can produce a total of eight transcripts (Kersey et al., 2018; Zerbino et al., 2018). T-1 is exclusively and abundantly expressed in the smooth muscle cells (SMCs) of normal adult vertebrates (Liu et al., 2017; Zhong et al., 2019) such that it is used as an early differentiation marker of SMCs (Lawson et al., 1997), and the differences in T-1 expression are mainly related to the regulation of SMC differentiation (Kato et al., 2019; Song et al., 2019).

### Characteristics of T-2

T-2, a homolog of SM22α, is also known as SM22 beta (SM22β), with an isoelectric point of 8.41 and a molecular weight of 22.39 kDa. The gene encoding T-2 in humans (TAGLN2) is located on chromosome 1q23.2 (Kim et al., 2017). It is regulated by alternative splicing, comprising seven exons that can produce five transcripts (Jo et al., 2018). T-2 localizes to multiple intracellular sites, including the cytoplasm, cell membrane and nucleus, and this difference in localization may be due to varying pathophysiological states (Lin et al., 2009). T-2 is the most widely distributed of the transgelin proteins; in addition to exhibiting high expression in SMCs and epithelial cells, it is also expressed in stem cells, bone marrow cells, and many organs (Kuo et al., 2011; Na et al., 2015; Meng et al., 2017; Yin et al., 2018). Furthermore, it is also strongly expressed in cancer tissues such as CRC (Zhang et al., 2010; Elsafadi et al., 2020).

## Involvement of Transgelins in CRC Metastasis

Transgelins, including T-1 and T-2, are expressed at different levels during CRC progression, and a summary of studies on the altered expression patterns of T-1 and T-2 in CRC metastasis is shown in Table 2.

**TABLE 2:** Summary of studies about altered level of transgelins in CRC metastasis.

Research design	Isoforms	Tissue for analysis	*Methodology	Altered level	Effects on CRC	References
Submucosal non-invasive CRC; submucosal invasive CRC	T-1	Whole tissue	P, T, I	Up in submucosal invasive CRC	Worse prognosis	Zhang et al., 2011
Poor prognosis; good prognosis (stage IV CRC)	T-1	Whole tissue	P, T	Up in poor prognosis	Worse prognosis	Kim et al., 2012
Node-positive CRC; node-negative CRC	T-1	Microdissected cells	P, T, I	Up in node-positive CRC	Worse prognosis	Lin et al., 2009
Node-positive CRC; node-negative CRC	T-1	Whole tissue	P, T	Up in node-positive CRC	Worse prognosis	Zhou H. M. et al., 2016
Early *COAD; normal mucosa	T-1	Whole tissue	P, T, I	Down in early COAD	Worse prognosis	Elsafadi et al., 2020
Late COAD; early COAD	T-1	Whole tissue	P, T, I	Up in late COAD	Worse prognosis	Elsafadi et al., 2020
Carcinoma; normal mucosa	T-2	Microdissected cells	P, I	Up in carcinoma	Worse prognosis	Zhang et al., 2010

*Methods used for analysis. P, Proteomics; T, transcriptomics; I, Immunohistochemistry. *COAD, Colon adenocarcinoma.

### Levels of T-1 in CRC Metastasis

T-1 has been acknowledged to be up-regulated in some solid tumors (Zhang et al., 2010, 2015; Ogut et al., 2020). The expression of T-1 was up-regulated in sera from patients with advanced disease, which could be caused by pathological hyperplasia of myofibroblasts and SMCs together with deeper tumor invasion into muscle layers (Peng et al., 2009). Down-regulating T-1 will weaken the metastatic ability of CRC, whereas restoring or increasing T-1 expression will increase the metastatic ability of CRC (Lee et al., 2010; Zhang et al., 2011). Furthermore, studies confirmed a positive correlation between T-1 level and lymph node metastasis by analyzing 24 microdissected human CRC specimens (Lin et al., 2009), and elevated expression of T-1 indicated a worse prognosis in advanced CRC (Kim et al., 2012). Zhou H. M. et al. (2016) observed that overexpressing transgelin in CRC cells led to an increase in the number and size of lung metastases in a mouse tail vein injection model, but attenuation of transgelin expression decreased both parameters in the same model. In addition, proteomic studies of T-1 frequently indicate up-regulation in aggressive late-stage disease (Elsafadi et al., 2020). Taken together, these data indicate that as the disease progresses, T-1 expression is positively associated with CRC metastasis.

### Levels of T-2 in CRC Metastasis

Some studies show that T-2 expression is positively related to lymph node and distant metastasis in CRC (Zhang et al., 2010). Colorectal villous adenoma, a precancerous disease, exhibits higher expression levels of T-2 (50% over baseline), suggesting that it is associated with CRC (Zhuo et al., 2012). In addition, there was an obvious correlation between T-2 expression and postoperative survival, and the mean survival of patients with low, moderate, and high levels of T-2 expression was 49.8 ± 13.6, 31.6 ± 19, and 11.4 ± 11 months, respectively (Zhang et al., 2010). Measuring T-2 expression in the tissues of 120 patients with CRC revealed that the survival in patients with low or no expression of T-2 was 20 months longer than that in patients with elevated expression (Zhuo et al., 2012). Consequently, T-2 may serve as a potential biomarker for predicting the progression and prognosis of CRC.

## The Mechanism of Transgelins in CRC Metastasis

**FIGURE 2:**
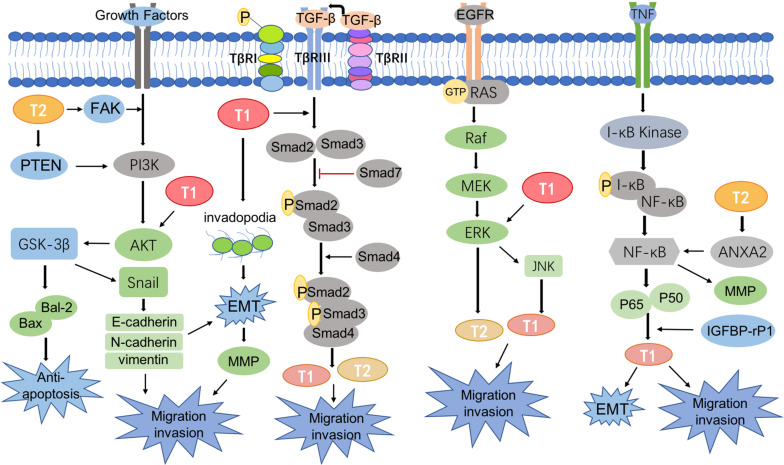
Signaling pathways involved in transgelin.

CRC metastasis is mediated by a complicated network of signaling pathways, many of which have been reported to involve T-1 and T-2, including the TGF-β/Smad pathway (Ali et al., 2010), phosphoinositide 3-kinase (PI3K)/phosphatase and tensin homolog (PTEN)/AKT pathway (Cai et al., 2014), KRAS-ERK signaling pathway (Eser et al., 2014), and NF-κB signaling pathway (Shi et al., 2020; Figure 2).

### PI3K/AKT Signaling Pathway

Many studies have proven the important role of AKT signaling in the metastasis and prognosis of CRC (Miller et al., 2020; Varga et al., 2020). The PI3K/AKT signaling pathway is a bridge that transduces extracellular activity into intracellular responses. The AKT pathway, which acts on intracellular targets, is activated by phosphorylating the loop at Thr308 and Ser473, which up-regulates T-1 levels and promotes CRC metastasis (Chen P. J. et al., 2019). Subsequently, studies showed decreased T-1 expression in CRC cells treated with AKT pathway inhibitors, and AKT pathway may promote CRC metastasis via up-regulation of T-1 (Zhou H. et al., 2016). By contrast, Chunhua et al. (2013) demonstrated that apigenin decreases AKT phosphorylation by up-regulating T-1 expression in mitochondria, which thus down-regulates MMP-9 activity to prevent the proliferation and metastasis of CRC cells. *In vitro* experiments also confirmed that apigenin inhibited tumor growth and lung and liver metastasis in a CRC model (Chunhua et al., 2013). Therefore, the role of T-1 in CRC remains controversial.

On the other hand, evidence has shown that T-2 can affect the migration and invasion of tumor cells by regulating the PI3K/AKT pathway (Pei et al., 2018). Liu et al. (2019) found that T-2 was located upstream of PTEN and directly interacted with it. Overexpression of T-2 could activate the PI3K/AKT/Gsk-3β pathway by interacting with PTEN as well as up-regulating bal-2 and down-regulating bax to inhibit apoptosis, leading to the increased migration and invasion of tumors (Liu et al., 2019). Focal adhesion kinase (FAK), a non-receptor tyrosine kinase found in virtually all mammalian cells, plays a role in cytoskeletal dynamics (Mitra et al., 2005; Frame et al., 2010). T-2 could directly interact with FAK and then activate the IGF1Rβ/PI3K/AKT pathway to increase the level of Snail1 expression and induce pathological EMT, which conveys invasive and migratory characteristic to tumor cells (Kim et al., 2018). Therefore, targeting T-1 and/or T-2 could be a strategy for treating CRC metastasis.

### TGF-β/Smad Signaling Pathway

TGF-β plays a multi-faceted role in regulating tumor cell growth and migration by acting as an inhibitor of cell proliferation in early stages of CRC but reversing course and exerting oncogenic activity during the later stages of CRC progression (Katz et al., 2016; Syed, 2016). Serum response factor (SRF) and transforming growth factor beta (TGF-β) work together to activate the T-1 promoter, which comprises SRF-binding CArG boxes, a Smad-binding element (SBE) and a TGF-β control element (TCE), and T-1 promoter activity is dependent on Smad signaling (Chen et al., 2003). Studies confirmed that TGF-β stimulated the binding of Smad3 to the chromatin containing the T-1 promoter and recruited and activated the transcription of specific target genes (Qiu et al., 2006). Some studies demonstrated that T-1 was positively associated with TGF-β, induced EMT, and promoted invadopodia formation; these changes resulted in significant increases in the migration capacity of tumor cells. Similarly, TGF-β-mediated metastasis disappeared when T-1 expression was inhibited (Chen Z. et al., 2019). In addition, the TGF-β/Smad pathway interacts with Wnt/β-catenin signaling, which is well-known to be associated with tumor metastasis to control T-1 expression (Shafer and Towler, 2009). Although T-1 is downstream of TGF-β, the effects of a TGF-β inhibitor on actin cytoskeleton changes were the same as those of T-1 depletion, and both approaches reduced CRC migration (Elsafadi et al., 2020), suggesting that T-1 is a novel target in CRC metastasis.

Additionally, T-2 expression was induced with TGF-β stimulation in a Smad4-dependent manner, which explained its role in CRC progression (Ali et al., 2010; Koudelkova et al., 2017).

### KRAS/ERK Signaling Pathway

KRAS gene mutations are the most frequently described in the majority of cancers (Aguilera and Serna-Blasco, 2018; Luo et al., 2019; Senturk et al., 2019), especially in CRC, and up to 40% of all patients have a known KRAS mutant gene (Aguilera and Serna-Blasco, 2018). RAS proteins exhibit GTPase activity; when Grab2 activates SOS, the GDP bound to RAS is exchanged for GTP (GTP-RAS). GTP-RAS then activates Raf, which binds to and phosphorylates MAPK; activated MAPK finally activates transgelin. Moreover, JNK is one of the important downstream branches of MAPK signaling (Wang et al., 2019), and Zhou H. et al. (2016) showed that JNK induced the overexpression of T-1. In addition, the TAGLN gene itself plays a critical role in regulating T-1 transcription through epigenetic modifications such as DNA methylation. Overexpression of T-1 leads to increased activation of RAS and ERK1/2, whereas its down-regulation results in a decrease in activated RAS (GTP-RAS) and downstream ERK1/2 activation (Park et al., 2014). These results indicate that T-1 may be a regulatory element in KRAS/ERK signaling.

Studies also found that KRAS mutations could up-regulate the expression and maintain the stability of T-2. It should be noted that other downstream effectors of KRAS may also be involved in the regulation of T-2. ERK interacts with amino acids 29–31 of T-2 and phosphorylates residue 145, which plays an important role in tumor metastasis (Sun et al., 2018; Senturk et al., 2019).

### NF-κB Signaling Pathway

NF-κB is a key promoter of tumorigenesis (Buhrmann et al., 2020). Tumor necrosis factor (TNF) binds to related receptors, predominantly activating I-κB kinase to allow the translocation of NF-κB into the cell nucleus and induce target gene expression (Yang et al., 2020). Braitsch et al. (2019) confirmed that the deletion of large tumor suppressor kinases 1 and 2 (LATS1/2) led to NF-κB activation and EMT, which was accompanied by up-regulated T-1 expression and ultimately promoted cell migration and invasion. Insulin-like growth factor binding protein-related protein-1 (IGFBP-rP1) was found to be up-regulated in CRC patients with lymph node metastasis CRC patients, who also exhibit up-regulated levels of T-1 (Ruan et al., 2010). Overall, IGFBP-rP1 exerted an inhibitory effect on cell motility and tumor metastasis in CRC by regulating EMT, possibly through different signaling pathways, such as the NF-κB, Wnt, and TGF-β signaling pathways, during tumor progression (Ruan et al., 2010; Rao et al., 2014; Zhu et al., 2015).

Other studies demonstrated that T-2 phosphorylates Annexin A2 (ANXA2) and activates NF-κB protein, further promoting tumor cell invasion and metastasis (Shi et al., 2020).

## Conclusion

At present, CRC metastasis is the main cause of cancer-related death worldwide (Engstrand et al., 2018; Rahbari et al., 2019). Commonly, surgical resection (Zellweger et al., 2018), radiotherapy (Petrelli et al., 2018), chemotherapy (Muneoka et al., 2018), targeted therapy (Li et al., 2019), and immunotherapy (Overman et al., 2018) are conducted in various combinations for the treatment of CRC patients. However, most CRC patients experience from disease recurrence and metastasis within 5 years (Luo et al., 2018). Molecular-targeted therapies are a promising treatment for CRC, especially metastatic CRC.

In this review, we focused on the relationships between T-1, T-2, and CRC metastasis. Transgelins are localized to different parts of cells, resulting in inconsistent expression levels of T-1 and T-2 during the development and progression of CRC. In the early stage of CRC, T-1 expression gradually decreases with tumor progression, suggesting that T-1 gene deletion is an important early event in tumor progression and is a diagnostic marker for CRC. However, when tumor cells are capable of invasion and metastasis, T-1 expression begins to rebound and continues to rise with the further progression of metastasis, and the expression levels of T-1 are positively correlated with worse prognosis in CRC patients with lymph node metastasis. However, unlike T-1, which has highly complex expression patterns and activities, T-2 always exhibits increased levels with the development and progression of CRC, especially in metastasis occurring in advanced CRC; the expression of T-2 in CRC metastatic tissues is significantly higher than that in tissues without metastasis and is positively associated with patient survival. However, there are few research results that are varied and controversial. These differences may be due to the fact that transgelin is expressed at multiple intracellular sites.

Importantly, transgelins not only adjust their expression to varying degrees during CRC progression but also participate in CRC metastasis via different signaling pathways. Most studies have shown that transgelins, including T-1 and T-2, interact with associated proteins to activate or inhibit signaling to regulate CRC metastasis. Outside the four signaling pathways mentioned in a previous article, a new study found that transgelin could bind to poly(ADP-ribose) polymerase-1 (PARP1) and regulate downstream genes, which are mainly involved in the Rho signaling pathway, initiate cytoskeletal remodeling, and induce CRC metastasis (Lew et al., 2020). A summary of transgelins about interacted proteins initiating feedback regulations to this signaling and the effects on CRC metastasis is shown in Table 3. Otherwise, the cartilage oligomeric matrix protein (COMP) interacts with transgelin in EMT to regulate cytoskeletal remodeling and promote malignant progression in CRC (Zhong et al., 2020). Since T-2 phosphorylation is closely related to cell movement, inhibiting T-2 phosphorylation may prevent cell migration and proliferation in CRC (Leung et al., 2011; Sun et al., 2018). Above all, transgelins are closely associated with metastasis in CRC and may be used as a target for the treatment of metastatic CRC.

**TABLE 3:** Summary of transgelins about feedback regulations to signaling pathways in CRC metastasis

Isoforms	Signaling pathway	Directly interacted	Downstream effectors	Effects on CRC	References
T-1	TGF-β/Smad	Smad3	Wnt3a, β-Catenin	Promote metastasis	Shafer and Towler, 2009
T-1	KRAS/ERK	ERK	JNK	Promote metastasis	Park et al., 2014
T-1	Rho/ROCK	PARP1	Rho GTPase	Promote metastasis	Lew et al., 2020
T-2	PI3K/AKT	PTEN, FAK	PI3K, AKT, Snail1	Promote metastasis	Kim et al., 2018; Liu et al., 2019
T-2	NF-κB	ANXA2	NF-κB	Promote metastasis	Shi et al., 2020

## Author Contributions

All authors listed have made a substantial, direct and intellectual contribution to the work, and approved it for publication.

## Conflict of Interest

The authors declare that the research was conducted in the absence of any commercial or financial relationships that could be construed as a potential conflict of interest.
