# Supplementary figures and images for: Transgelins: Cytoskeletal Associated Proteins Implicated in the Metastasis of Colorectal Cancer
Source: Front Cell Dev Biol. 2020 Oct 7;8:573859. doi: 10.3389/fcell.2020.573859 (PMC7575706; doi:10.3389/fcell.2020.573859)

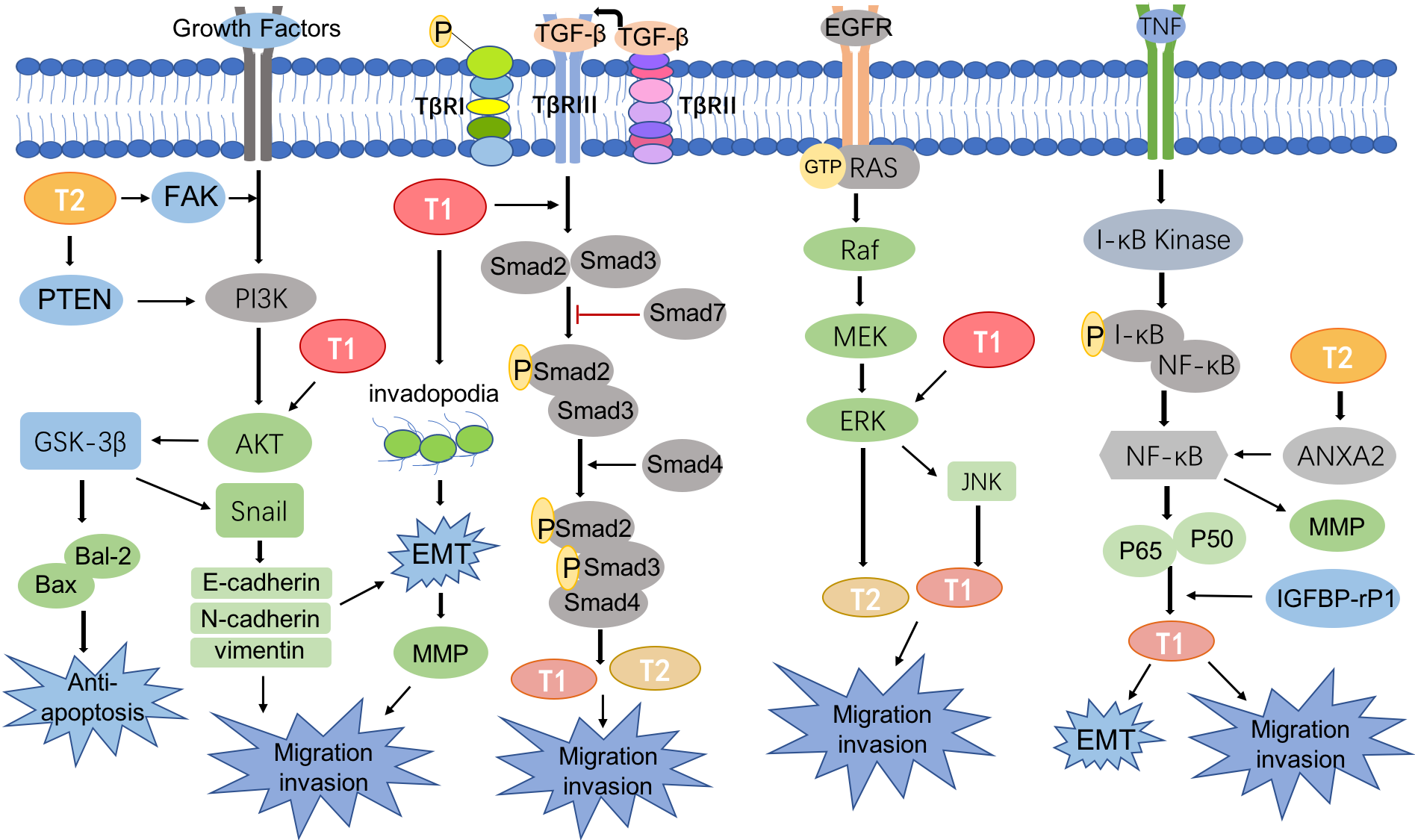

Supplement: Supplementary file 1 [file Data_Sheet_1.ZIP › μ£║σê╢σ¢╛-2020.8.25Σ┐«.jpg]

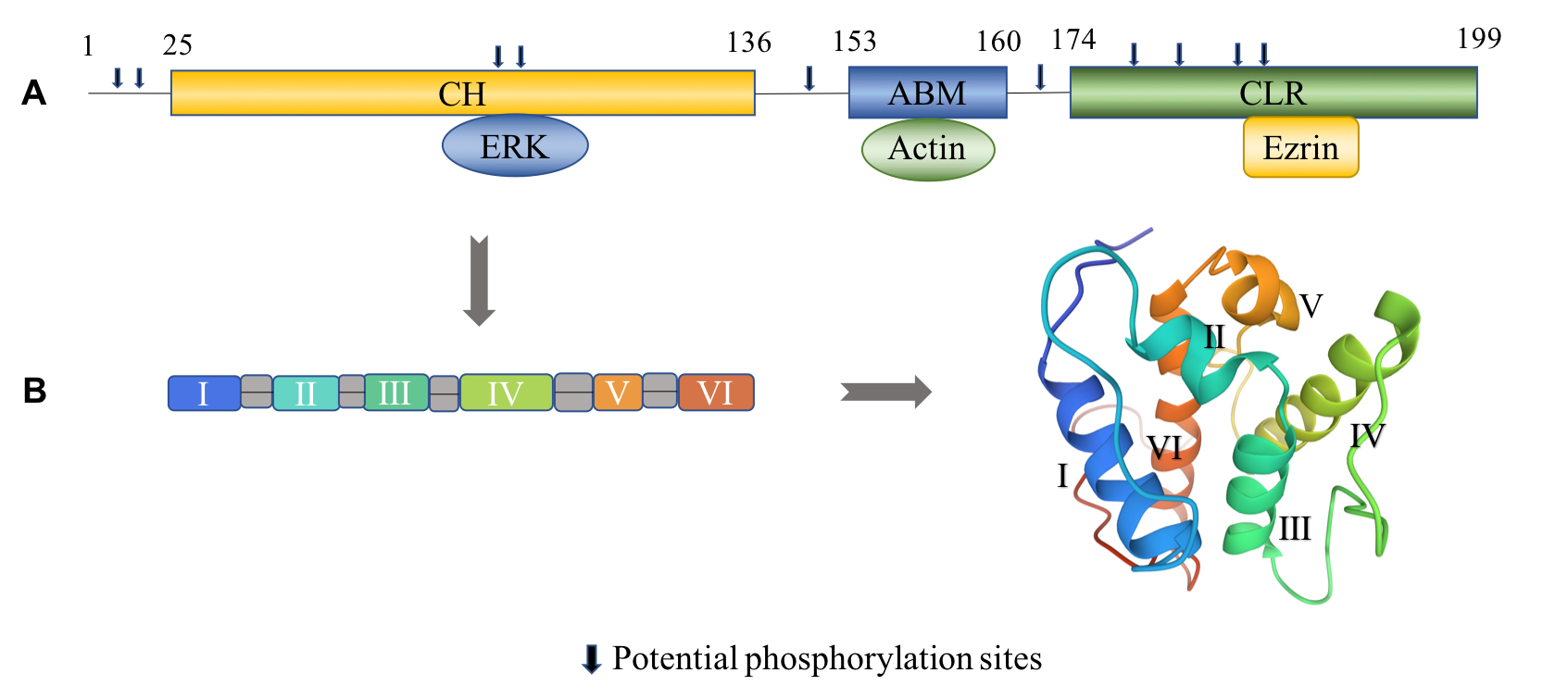

Supplement: Supplementary file 1 [file Data_Sheet_1.ZIP › Θ¬¿μ₧╢Φ¢ïτÖ╜τ╗ôμ₧äσ¢╛2020.8.27.jpg]
